# Uterine Cervical Cancer Treatment and Outcomes Among Medicare-Insured Patients in the United States

**DOI:** 10.3390/healthcare14203488

**Published:** 2026-10-17

**Authors:** Diego B. Lopez-Gonzalez, Madeleine Sertic, Chaya S. Moskowitz, Robert G. Rasmussen, Aoife Kilcoyne, Avinash Kambadakone, Wui-Jin Koh, Susanna I. Lee

**Affiliations:** 1 Department of Radiology, Mass General Brigham, Harvard Medical School, Boston, MA 02114, USAakambadakone@mgh.harvard.edu (A.K.); 2 Joint Department of Medical Imaging, University Health Network, Mount Sinai Hospital and Women’s College Hospital, University of Toronto, Toronto, ON M5G 24C, Canada; 3 Department of Epidemiology and Biostatistics, Memorial Sloan Kettering Cancer Center, New York, NY 10065, USA; 4 National Comprehensive Cancer Network, Plymouth Meeting, PA 19462, USA; wjaakoh1@comcast.net

**Keywords:** cervical cancer, socioeconomic status, health disparities, Medicare, cancer-specific survival

## Abstract

**Highlights:**

**What are the main findings?**

- In this retrospective cohort study of 14,552 Medicare-insured women with cervical cancer, lower socioeconomic status was significantly associated with reduced likelihood of receiving primary treatment and poorer long-term cancer-specific survival in multivariable analysis.
- Black patients were less likely and Hispanics slightly more likely to receive treatment compared to Whites.

**What are the implications of the main findings?**

- Even among those with insurance coverage, variations in treatment rates and cancer-specific outcomes were associated with socioeconomic status and race.
- In uterine cervical cancer treatment, medical insurance does not suffice to remove the social determinants of health.

**Abstract:**

Background/Objectives: Although approximately 90% of the United States population is now covered by medical insurance, variations in cancer treatment rates and survival persist even among the insured. Medicare offers a uniformly insured cohort where the underlying factors for this variability can be studied. This study evaluates whether nonclinical societal factors are associated with receipt of treatment and survival among Medicare-insured patients with cervical cancer. Methods: This retrospective cohort study used linked SEER-Medicare data from 2000 to 2018 to identify women aged 65 years and older with a first primary diagnosis of uterine cervical cancer. Patients without complete Medicare Parts A and B coverage were excluded. Exposures extracted were race, socioeconomic status (Yost index), and geography relative to healthcare (central metro vs. metro adjacent vs. rural). Outcome measures were primary treatment (surgery, chemotherapy, or radiotherapy within 6 months of diagnosis), cancer-specific survival, and overall survival. Data were analyzed using multivariable regression models. Results: Among 14,552 patients (mean age, 71 years [SD, 12]; 62.8% White, 17.9% Black, 12.0% Hispanic), 11,215 (77.1%) received primary treatment. Higher socioeconomic status was associated with a higher likelihood of receiving treatment (adjusted prevalence ratios [PR] of 1.12; 95% CI, 1.08–1.17 for the highest and 1.09; 95% CI, 1.06–1.14 for the second to highest quintiles respectively) and lower cancer-specific mortality (adjusted hazard ratio [HR], 0.81; 95% CI, 0.68–0.96 for the highest and 0.83; 95% CI, 0.71–0.97 for the second to highest quintiles respectively). Black patients were less likely to be treated (PR, 0.94; 95% CI, 0.91–0.98), although this did not translate into worse survival rates. Hispanic patients were slightly more likely to receive treatment (PR, 1.03; 95% CI, 1.00–1.06) and showed lower cancer-specific mortality (HR, 0.79; 95% CI, 0.67–0.94). Geography was not associated with treatment in multivariable analysis. Conclusions: In this cohort of Medicare-insured women with cervical cancer, variations in treatment rates and cancer-specific outcomes were associated with socioeconomic status and race, despite health insurance coverage. These findings highlight the need for efforts toward structural interventions to address the social determinants of health.

## 1. Introduction

Uterine cervical cancer disproportionately kills the underserved and is the leading cause of cancer death in women in 37 countries, predominantly in sub-Saharan Africa, Southeast Asia, and South America [1,2]. In the United States, approximately 4000 women die from cervical cancer each year, with this figure remaining intractably stable in the past five years [3]. This is in the context of ongoing declines in disease incidence during this same period attributable to widespread screening and human papillomavirus vaccination efforts [4,5]. Moreover, concurrent chemoradiotherapy as the standard of care for locoregional disease, as well as advances in minimally invasive surgical techniques, and the introduction of targeted systemic therapies such as immune checkpoint inhibitors and antibody–drug conjugates for advanced and recurrent disease have all improved survival rates [1,6]. Thus, cervical cancer deaths in the United States, analogous to the rest of the world, affect the medically underserved, those who have not been adequately screened or treated.

Multiple large cohort studies and previous national database analyses have shown that uninsured and Medicaid-insured patients with cervical cancer are less likely to receive timely or guidance-concordant treatment and are more likely to die from the disease [7,8,9]. Healthcare policies, such as the Affordable Care Act, have been undertaken with the hope that expanding adequate medical insurance coverage to a greater proportion of the population would mitigate or remove these access barriers. Yet even in settings where universal healthcare is available, variability in cancer outcomes continues to be described [10,11].

Cervical cancer treatment is resource- and time-intensive, both for the patient and the healthcare system. It requires specialized oncologic facilities and expertise, not widely or uniformly available, even in the developed world [12]. Definitive chemoradiotherapy for locoregionally advanced disease takes about 7–8 weeks and can require up to 40 visits during active treatment. Surgical management for early stage disease involves a hospital stay, which may be followed by adjuvant chemotherapy adding several weeks and multiple outpatient visits. [13] Thus, while medical insurance makes treatment financially feasible, it may not suffice in overcoming all these access barriers.

We hypothesize that, given the complexities of multimodality therapy involved in treating cervical cancer, medical insurance alone is unlikely to overcome the many nonclinical societal barriers to healthcare. This study asks whether variables such as race, socioeconomic status or geography are associated with access to cervical cancer treatment in patients who have comprehensive medical insurance. We confine our analysis to patients covered by Medicare Parts A and B, which insures medically necessary prevention and treatment services in both the inpatient and outpatient settings throughout the country. The relationship of the societal as well as the conventional clinical variables with long-term patient outcomes, i.e., cancer-specific and overall survival, is also estimated.

## 2. Materials and Methods

### 2.1. Study Cohort

**Figure 1:**
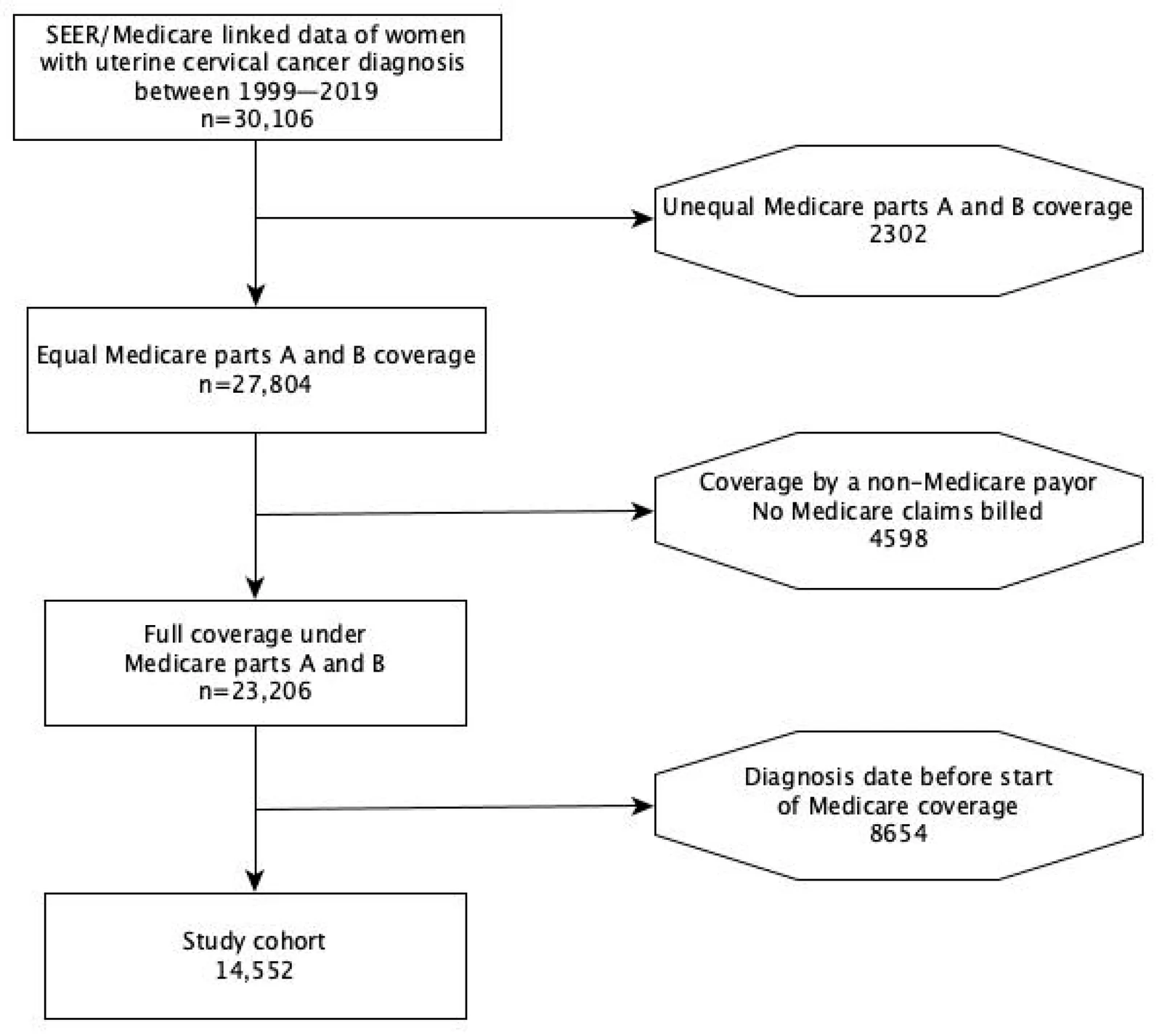
Cohort derivation.

The linked SEER-Medicare files, the Surveillance, Epidemiology, and End Results (SEER)/Medicare linked database [14] encompassing data on cancer patients collected at multiple sites, were the source for this institutional review-board-approved retrospective cohort study. The demographic and clinical data available on patients of any age presenting with a uterine cervical cancer diagnosis in the linked Medicare files spanned 2000 to 2018. All patients included in the cohort are eligible for cancer treatment under consensus guidelines. Figure 1 summarizes our cohort derivation. To capture the full spectrum of billed care and to limit this study to only the fully Medicare-insured population, patients with unequal Medicare Parts A and B coverage or in whom no Medicare claims were submitted, presumably because they were insured by another party, were excluded. Patients with concurrent active diagnosis and/or treatment for a second cancer within 2 years preceding or following the presentation were excluded.

### 2.2. Independent Variables

Among the nonclinical societal variables collected were race, the Yost index of socioeconomic status in quintiles, and geography (i.e., central metro, metro adjacent, not metro adjacent/rural). Yost index is a census tract-level measure of the neighborhood socioeconomic status of patients developed by the National Cancer Institute, categorized in quintiles [15]. Among the standard clinical variables, we extracted age and stage at diagnosis (localized, regional [≥FIGO stage IIB], advanced [≥FIGO stage IV]), cancer histology, comorbidities summarized by the Charlson Comorbidity Index, and advanced imaging use. Charlson Comorbidity Index quantifies the burden of comorbid diseases by combining weighted scores to a set of specific chronic conditions, with higher total scores correlating with increased risk of mortality [16]. Inclusion of this index adjusts for clinical factors associated with treatment indication and/or patient outcome. Advanced imaging was defined as CT, MRI and PET.

Imaging inpatient and outpatient claims were extracted from Medicare claims files, including the Medicare Beneficiary Summary Files (MSBF), MedPAR files, National Claims History (NCH) files, and Medicare outpatient claims files. Imaging claims were identified using relevant ICD-9 and ICD-10 codes for MedPAR files, CPT codes for NCH files, and Healthcare Common Procedure Coding System (HCPCS) codes for outpatient files for claims dated within 6 months of the date of diagnosis.

### 2.3. Outcome Variables

Primary treatment administration was defined as cancer-specific surgery, chemotherapy or radiation therapy administration within 6 months of diagnosis. Consensus guidelines and practice do not specify a timeline for initiating appropriate therapy, so the 6-month timeframe was chosen as a conservative timeframe within which indicated treatment would be performed in most patients. Multimodality therapy is the norm for many of the uterine cancer stages, and the available data were not sufficiently granular to define a subgroup of only the “treatment indicated and appropriate” patients. Death from all causes and specifically from cervical cancer was also collected.

### 2.4. Statistical Analysis

The Charlson Comorbidity Index was calculated using an algorithm developed by Stagg 2017 [17].

To test for associations with treatment for cervical cancer, log-binomial models with robust variance estimates were used to estimate prevalence ratios and corresponding 95% confidence intervals (CI).

Survival analyses were done using the time since cervical cancer diagnosis as the time scale. Time at risk started at 180 days after cervical cancer diagnosis. Only patients who were alive and under observation at the 180-day landmark were included in the survival analysis. This approach specifically does not attribute survival time during the treatment ascertainment period to a treatment group based on future treatment information and thus avoids immortal time bias. For overall survival, patients were considered at-risk until the date of death from any cause, and patients alive as of 31 December 2018 were censored. Kaplan–Meier estimates were used to estimate overall survival, and Cox proportional hazards regression was used to evaluate associations and estimate hazard ratios (HRs). The cumulative incidence of cervical cancer-specific survival was estimated using a non-parametric estimate treating deaths due to causes other than cervical cancer as a competing risk [18]. Factors associated with cervical cancer-specific survival were evaluated using cause-specific Cox models, censoring patients who died of other causes or were alive as of 31 December 2018. Multivariable models were constructed including variables that were predetermined to be clinically important. Patients who were missing data for any of the included variables were excluded from the models. The proportional hazards assumption was assessed for all Cox regression models using Schoenfeld residual-based tests. No evidence of meaningful departures from proportionality was observed.

All models converged successfully. We assessed potential multicollinearity among model covariates using variance inflation factors (VIFs). All VIF values were below 2.0 (range, 1.03–1.65), indicating minimal collinearity among the included predictors.

All tests were two-sided 0.05-level tests. Analyses were done in Stata 18 for Windows (StataCorp, LLC., College Station, TX, USA).

## 3. Results

The study cohort comprises 14,552 patients diagnosed with uterine cervical cancer between 2000 and 2018. (Table 1). A total of 11,215 (77.1%) received primary treatment. Median follow-up time was 8.2 years (range 2.2–21.0 years). Median survival after diagnosis was 3.5 years (95% CI: 3.3, 3.7). Overall survival at 5 years was 29% (95%CI: 28%, 31%).

To offer transparency regarding the composition of the analytic cohort and address the potential for selection bias associated with excluding patients with missing covariate data, Table S1 reports the characteristics of participants with complete versus missing covariate information. Missing covariate data, specifically stage at diagnosis, was considerably more common among participants diagnosed before 2004 (~35%) than among those diagnosed in 2004 or later (<10%). This pattern is driven largely by missing information on stage at diagnosis, which represents the primary source of covariate missingness in our analytic dataset.

Table 2 summarizes the findings of the multivariate model where the outcome is whether the patient underwent primary treatment. Among the nonclinical societal variables, socioeconomic status demonstrated a clear association, with treatment progressively less likely with category decreases of the Yost index (Yost 5 PR 1.12 [95% CI, 1.08–1.17]; Yost 4 PR 1.09 [95% CI, 1.06–1.14]; Yost 3 PR 1.08 [95% CI, 1.06–1.13]; Yost 2 PR 1.08 [95% CI, 1.04–1.12]). Blacks were less likely (PR, 0.94; 95% CI, 0.91–0.98) and Hispanics were more likely (PR, 1.03; 95% CI, 1.00–1.06) to receive treatment. Of note, we did not observe significant relationships between geography or comorbidities on whether a patient received treatment. Among the clinical variables, successive increase in stage was associated with lesser likelihood of treatment. Atypical histologies (i.e., not squamous or adenocarcinoma) were also less likely to be treated (PR 0.94; 95% CI, 0.90–0.98). Imaging with MRI (PR 1.04; 95% CI, 1.01–1.06) and PET (PR 1.13; 95% CI, 1.11–1.16), standardly used for chemoradiation treatment planning, showed a positive association with therapy; however, CT (PR 1.00; 95% CI, 0.98–1.02), more generally used for staging and follow-up, did not [19,20].

**Figure 2:**
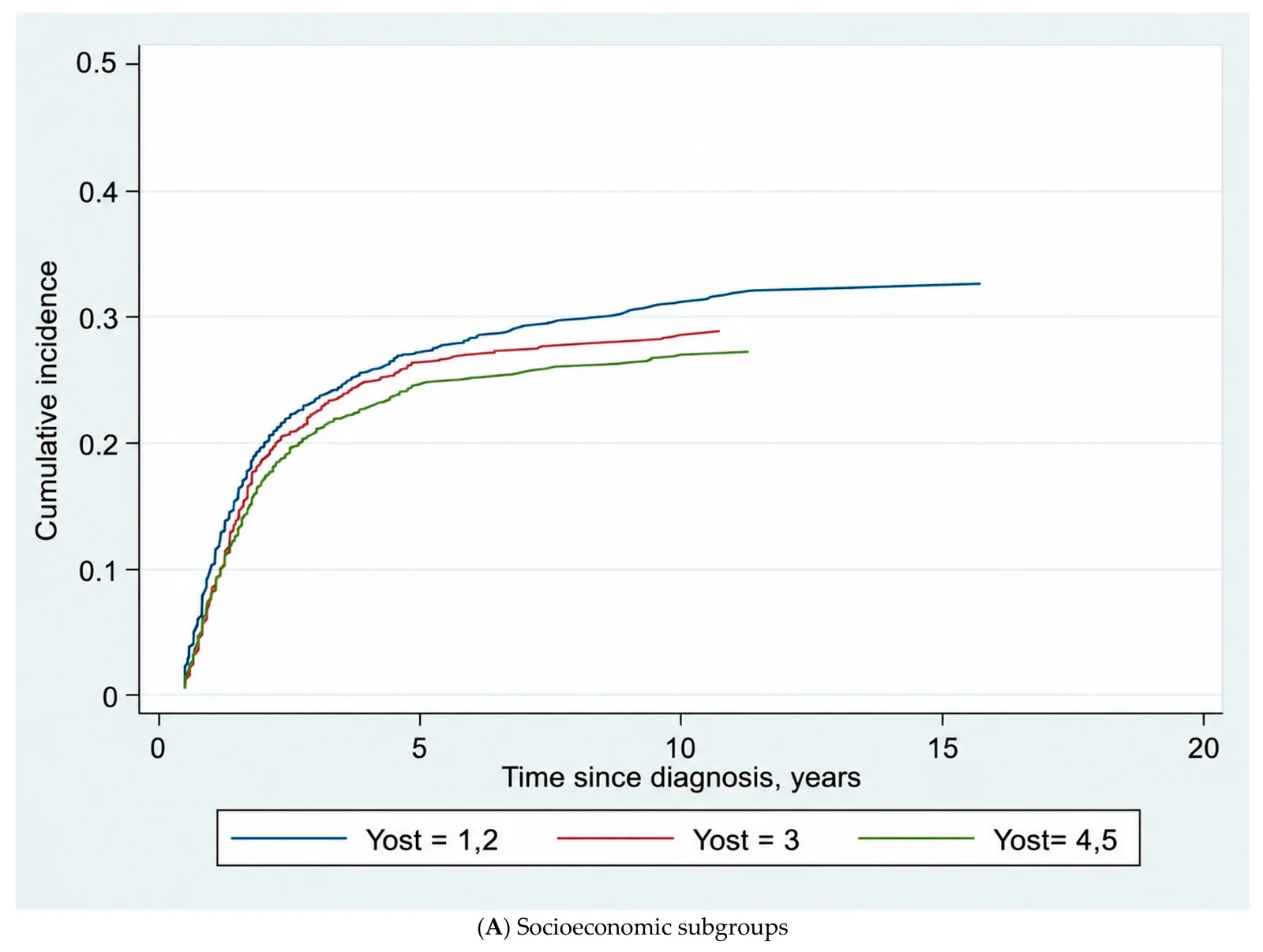

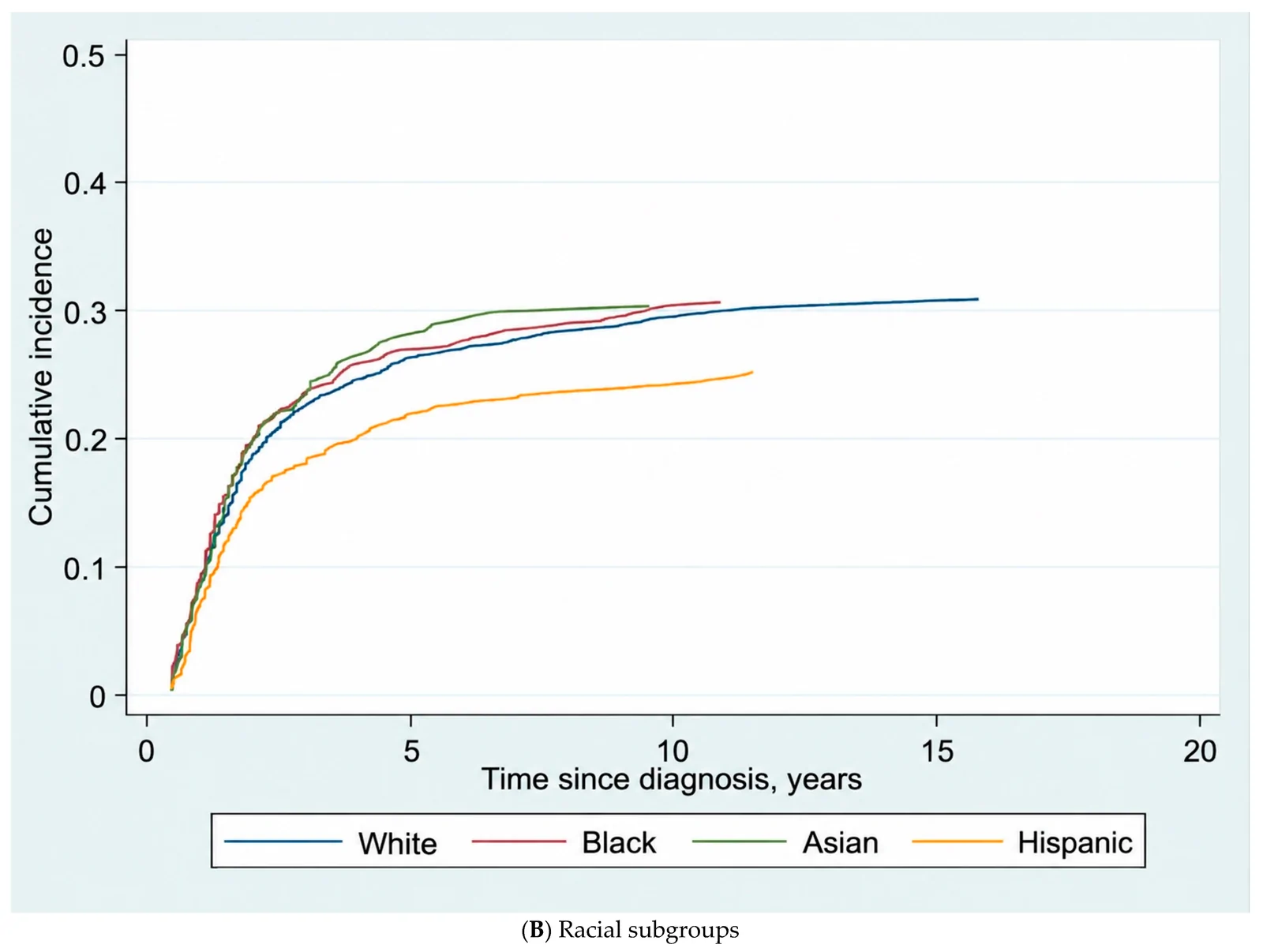
Long-term cancer-specific mortality. (**A**) By Yost index category and (**B**) by race.

Table 3 summarizes the factors associated with cancer-specific mortality in the multivariate model that controls for treatment status. Among the nonclinical societal variables (Figure 2), socioeconomic status was associated with cancer-specific mortality, with the successive lower quintiles demonstrating higher mortality (Yost 5 PR 0.81 [95% CI, 0.68–0.96]; Yost 4 PR 0.83 [95% CI, 0.71–0.97]; Yost 3 PR 0.86 [95% CI, 0.74–1.00]; Yost 2 PR 0.90 [95% CI, 0.78–1.04]). Hispanics demonstrated better cancer-specific mortality outcomes (PR 0.79; 95% CI, 0.67–0.94). In Blacks, poorer cancer-specific outcomes could not be demonstrated once treatment status was accounted for (PR 0.97; 95% CI, 0.84–1.11). With geography, patients in greater metro areas demonstrated a slightly lower likelihood of cancer-specific mortality (PR 0.88; 95% CI, 0.79–0.99) compared to those in central metro or rural locations. Among the clinical variables, expectedly, increasing tumor stage, non-squamous histology, greater burden of comorbidities, and primary treatment not occurring were all associated with worse outcomes. CT, used more often in evaluating patients with suspected advanced stage disease, was associated with poorer outcomes.

Table 4 summarizes the factors associated with overall survival outcomes in the multivariate model. Cancer stage (regional tumor spread PR 1.89 [95% CI, 1.74–2.06]; distant tumor spread PR 3.80 [95% CI, 3.41–4.24]); comorbidities (Charlson 0 PR 0.87 [95% CI, 0.28–2.71]; Charlson 1 and 2 PR 0.27 [95% CI, 0.20–0.38]; Charlson 3 and 4 PR 0.39 [95% CI, 0.33–0.47]; Charlson 5–9 PR 0.68 [95% CI, 0.63–0.73]); and whether primary treatment occurred (PR 0.52; 95% CI, 0.47–0.56) were all associated with overall mortality. No significant associations were seen with socioeconomic status or patient geography.

## 4. Discussion

Our retrospective multicenter cohort study shows that, even among the adequately insured, relationships between nonclinical factors and healthcare inequalities are identified in women diagnosed with cervical cancer. The most important factor is lower socioeconomic status, which is reliably associated with a lower likelihood of primary treatment and lower cancer-specific survival. Concerning race, Blacks are less likely to be treated; however, our study could not demonstrate worse long-term outcomes once treatment occurred. Hispanics are more likely to be treated and show slightly better cancer-specific survival. Our findings indicate that, even in populations with adequate medical insurance, variation in healthcare access and outcomes persists and that efforts to identify and address these social, economic and cultural factors should be pursued.

Cohort analyses and survey studies have shown that, beyond insurance coverage, other barriers contribute to healthcare inequities [21,22,23,24,25,26]. Low socioeconomic status patients are less likely to have the financial resources to overcome logistical barriers such as long-distance travel and stays for treatment visits, mustering help for caregiver responsibilities at home, and out of pocket copay costs. Such deficits result in environmental instabilities that can shift decision-making toward immediate needs at the expense of long-term health goals. Lower socioeconomic status is also associated with less access to education and digital information, limiting health literacy and the ability of the patient to participate in shared decision-making.

Ample literature supports our observation that Black patients are less likely to receive treatment for their cancer. This difference persists even in the context of programs to help patients with low health literacy navigate the healthcare system [27,28]. This is thought to be driven in part by both a systemic bias among the providers and the patients’ mistrust of the healthcare system based on past experiences. Black cancer patients are more likely to report negative care episodes, such as dismissal by providers, exclusion from decision-making, and overt discrimination. [29].

Multiple large population-based studies indicate that Hispanic women with cervical cancer show, on average, better treatment outcomes compared to non-Hispanic White women [30,31]. Past findings agree with our observation that Hispanic women show a decreased hazard ratio of cervical cancer-specific mortality relative to non-Hispanic Whites when factors such as insurance and socioeconomic status and the relevant clinical variables (e.g., stage, comorbidities) are controlled for. Defining whether this ethnic advantage is associated with biologic (e.g., genetics, diet) or cultural (e.g., social structure, value system) factors could be a topic of future investigations.

There is evidence that cervical cancer patients in rural geographic areas are less likely to receive appropriate and/or timely care and experience poorer survival outcomes [32,33]. However, in our study population, unlike those previously analyzed by being older and having health insurance, such associations were not reliably seen. Older patients are less likely to be working and thus less likely to be faced with a requirement to give up a salary to travel to and stay at distant locations to obtain care. It is also possible that the healthcare coverage offers hope and incentivizes the patient to overcome these logistical issues. Whether health insurance suffices to overcome geographic barriers to care in the younger cancer patient would be of interest.

Policy interventions at a societal level have proven effective in the past in improving outcomes of cancer patients. Population-based studies have shown that expanding health insurance to the previously uninsured is associated with improved treatment access and outcomes in cervical cancer [34,35]. In the United States, expansion of Medicaid under the Affordable Care Act resulted in more timely treatment for women, with gynecologic cancers, including cervical cancer, having the most pronounced benefits among low-income and minority populations. Our study suggests that, despite the successes of the screening and treatment efforts, cervical cancer deaths persist because underlying socioeconomic and racial barriers to care persist, even for the patient with adequate health insurance. Efforts toward decreasing cervical cancer deaths will require that these nonclinical societal factors be better defined and addressed at a community, institutional, or public policy level.

Our study has several important limitations. As a retrospective observational analysis, we identify factors associated with outcomes of interest, but causality is not demonstrated. Additionally, because of the limitations of the SEER/Medicare database that has been collected, we cannot be more specific about the nature of the socioeconomic and racial factors identified. Future studies to define these in greater detail are required before solutions toward mitigating them can be formulated. While only cancer-specific treatment within a 6-month period of presentation was analyzed and the Charlson Comorbidity Index included in the model adjusted for confounders, we have not performed a subgroup analysis of only those individuals labeled as medically indicated for uterine cancer therapy. Thus, we did not verify that each therapy or combination of therapies performed was individually appropriate for the patient’s stage and clinical condition. The data included in SEER/Medicare were not sufficiently granular to define that subgroup. Our Medicare-insured population is older and carries more comorbidities than the typical cervical cancer patient, who is most commonly diagnosed between ages 35 and 44 years [36]. Finally, our cohort is drawn from a span of nearly two decades during which time much has changed in cervical cancer care both clinically and socially.

In conclusion, among uterine cervical cancer patients with adequate healthcare coverage under Medicare, variation in healthcare access and outcomes continues to be seen and is associated with nonclinical features. Those with lower socioeconomic status are less likely to be treated and demonstrate worse cancer-specific outcomes. Blacks are less likely to be treated, whereas Hispanics are more likely to be treated and experience better survival outcomes. While policy strides toward universal healthcare coverage have yielded progress, more research and interventions in the public health and socioeconomic domain are necessary to fully address the persistent inequities in cervical cancer care.

## Figures and Tables

**Table 1:** Baseline demographic, sociologic, and clinical characteristics of uterine cervical cancer patients who received or did not receive primary treatment.

	Treated		
	No n (%)	Yes n (%)	Total n (%)
Study cohort	3337 (22.9%)	11,215 (77.1%)	14,552 (100.0%)
Age, yrs	75.99 (11.89)	69.33 (11.89)	70.85 (12.21)
Age			
18–29	9 (0.3%)	51 (0.5%)	60 (0.4%)
30–44	72 (2.2%)	592 (5.3%)	664 (4.6%)
45–64	405 (12.1%)	2259 (20.1%)	2664 (18.3%)
65–74	993 (29.8%)	5011 (44.7%)	6004 (41.3%)
75+	1858 (55.7%)	3302 (29.4%)	5160 (35.5%)
Year of diagnosis			
Before 2004	886 (26.6%)	2621 (23.4%)	3507 (24.1%)
2004+	2451 (73.4%)	8594 (76.6%)	11,045 (75.9%)
Race			
White	2055 (61.6%)	7077 (63.1%)	9132 (62.8%)
Black	724 (21.7%)	1884 (16.8%)	2608 (17.9%)
Asian	245 (7.3%)	787 (7.0%)	1032 (7.1%)
Hispanic	305 (9.1%)	1443 (12.9%)	1748 (12.0%)
Unknown	8 (0.2%)	24 (0.2%)	32 (0.2%)
Geography			
Metro	897 (26.9%)	2819 (25.1%)	3716 (25.5%)
Adjacent to metro	2172 (65.1%)	7771 (69.3%)	9943 (68.3%)
Not adjacent to metro	235 (7.0%)	553 (4.9%)	788 (5.4%)
Unknown	33 (1.0%)	72 (0.6%)	105 (0.7%)
Yost Index *			
1	712 (21.3%)	1942 (17.3%)	2654 (18.2%)
2	440 (13.2%)	1772 (15.8%)	2212 (15.2%)
3	387 (11.6%)	1577 (14.1%)	1964 (13.5%)
4	375 (11.2%)	1665 (14.8%)	2040 (14.0%)
5	322 (9.6%)	1600 (14.3%)	1922 (13.2%)
Unknown	1101 (33.0%)	2659 (23.7%)	3760 (25.8%)
Charlson Index ^			
0	49 (1.5%)	164 (1.5%)	213 (1.5%)
1–2	144 (4.3%)	628 (5.6%)	772 (5.3%)
3–4	309 (9.3%)	1112 (9.9%)	1421 (9.8%)
5–9	1070 (32.1%)	3355 (29.9%)	4425 (30.4%)
10+	1747 (52.4%)	5912 (52.7%)	7659 (52.6%)
Unknown	18 (0.5%)	44 (0.4%)	62 (0.4%)
Histology			
Squamous cell	1930 (57.8%)	7482 (66.7%)	9412 (64.7%)
Adenocarcinoma	554 (16.6%)	2222 (19.8%)	2776 (19.1%)
Other	853 (25.6%)	1511 (13.5%)	2364 (16.2%)
Stage			
Localized	589 (17.7%)	3677 (32.8%)	4266 (29.3%)
Regional	1252 (37.5%)	4550 (44.4%)	5802 (42.7%)
Distant	733 (22.0%)	1479 (14.4%)	2212 (16.3%)
Unstaged/unknown	763 (22.9%)	1509 (13.5%)	2272 (15.6%)
Advanced imaging			
No	934 (28.0%)	2632 (23.5%)	3566 (24.5%)
Yes	1686 (50.5%)	6111 (54.5%)	7797 (53.6%)
Unknown	717 (21.5%)	2472 (22.0%)	3189 (21.9%)
MRI			
No	2255 (67.6%)	6865 (61.2%)	9120 (62.7%)
Yes	365 (10.9%)	1878 (16.7%)	2243 (15.4%)
Unknown	717 (21.5%)	2472 (22.0%)	3189 (21.9%)
PET			
No	2039 (61.1%)	5616 (50.1%)	7655 (52.6%)
Yes	581 (17.4%)	3127 (27.9%)	3708 (25.5%)
Unknown	717 (21.5%)	2472 (22.0%)	3189 (21.9%)
CT			
No	1291 (38.7%)	4329 (38.6%)	5620 (38.6%)
Yes	1329 (39.8%)	4414 (39.4%)	5743 (39.5%)
Unknown	717 (21.5%)	2472 (22.0%)	3189 (21.9%)
Vital status			
Alive	313 (9.4%)	4199 (37.4%)	4512 (31.0%)
Deceased from cervical cancer	2037 (61.0%)	2585 (23.0%)	4622 (31.8%)
Deceased from other causes	957 (28.7%)	1674 (14.9%)	2631 (18.1%)
Deceased from unknown causes	30 (0.9%)	2757 (24.6%)	2787 (19.2%)

Continuous numbers are summarized with means (standard deviations). * Successive increase in quintile indicates higher socioeconomic status. ^ Higher index is associated with greater morbidity burden. 0 category includes all patients for whom no comorbidities were reported and thereby includes those with missing data.

**Table 2:** Associations of demographic, sociologic, and clinical features with primary treatment administration of uterine cervical cancer.

	Prevalence Ratio (PR)	95% CI	*p* Value ^~^
Age			
18–30	1.00		
30–45	0.86	0.82 0.91	<0.001
45–65	0.84	0.80 0.87	<0.001
65–75	0.82	0.79 0.86	<0.001
75+	0.66	0.63 0.69	<0.001
Year of diagnosis	1.00	0.99 1.00	
Race			
White	1.00		
Black	0.94	0.91 0.98	<0.001
Asian	1.00	0.95 1.04	<0.001
Hispanic	1.03	1.00 1.06	<0.001
Geography			
Metro	1.00		
Adjacent to metro	1.02	1.00 1.05	0.058
Not adjacent to metro	0.95	0.90 1.01	0.081
Unknown	0.99	0.86 1.13	0.816
Yost Index *			
1	1.00		
2	1.08	1.04 1.12	<0.001
3	1.08	1.04 1.13	<0.001
4	1.09	1.06 1.14	<0.001
5	1.12	1.08 1.17	<0.001
Charlson Index ^			
0	0.88	0.56 1.36	0.554
1–2	1.00	0.95 1.05	0.906
3–4	0.98	0.94 1.01	0.237
5–9	0.98	0.95 1.01	0.121
10+	1.00		
Histology			
Squamous cell	1.00		
Adenocarcinoma	1.01	0.99 1.04	0.288
Other	0.94	0.90 0.98	0.003
Stage			
Localized	1.00		
Regional	0.91	0.89 0.93	<0.001
Distant	0.79	0.76 0.82	<0.001
MRI			
No	1.00		
Yes	1.04	1.01 1.06	0.005
PET			
No	1.00		
Yes	1.13	1.11 1.16	<0.001
CT			
No	1.00		
Yes	1.00	0.98 1.02	0.246

7052 observations were included in the complete case model. * Successive increase in quintile indicates higher socioeconomic status. ^ Higher index is associated with greater morbidity burden. ^~^ *p* values < 0.05 considered significant.

**Table 3:** Associations of demographic, sociologic, and clinical features with uterine cervical cancer-specific mortality.

	Hazard Ratio	95% CI	*p* Value
Age			
18–30	1.00		
30–45	0.87	0.32 2.42	0.83
45–65	0.77	0.29 2.09	0.67
65–75	0.79	0.30 2.13	0.70
75+	0.98	0.36 2.64	0.98
Year of diagnosis	1.00	0.98 1.01	0.80
Race			
White	1.00		
Black	0.97	0.84 1.11	0.67
Asian	0.93	0.78 1.12	0.51
Hispanic	0.79	0.67 0.94	0.01
Geography			
Metro	1.00		
Adjacent to metro	0.88	0.79 0.99	0.03
Not adjacent to metro	1.18	0.96 1.43	0.12
Unknown	1.40	0.89 2.19	0.14
Yost Index *			
1	1.00		
2	0.90	0.78 1.04	0.16
3	0.86	0.74 1.00	0.07
4	0.83	0.71 0.97	0.03
5	0.81	0.68 0.96	0.02
Charlson Index			
0 ^	1.23	0.31 4.98	0.76
1–2	0.28	0.17 0.45	<0.001
3–4	0.45	0.34 0.58	<0.001
5–9	0.71	0.63 0.79	<0.001
10+	1.00		
Histology			
Squamous cell	1.00		
Adenocarcinoma	1.24	1.09 1.40	<0.001
Other	1.31	1.13 1.52	<0.001
Stage			
Localized	1.00		
Regional	2.66	2.32 3.06	<0.001
Distant	6.28	5.35 7.37	<0.001
MRI			
No	1.00		
Yes	0.99	0.88 1.12	0.89
PET			
No	1.00		
Yes	0.97	0.87 1.09	0.63
CT			
No	1.00		
Yes	1.20	1.07 1.33	<0.001
Treated			
No	1.00		
Yes	0.38	0.34 0.43	<0.001

5899 observations were included in the complete case model. * Successive increase in quintile indicates higher socioeconomic status. ^ Higher index is associated with greater morbidity burden.

**Table 4:** Associations of demographic, sociologic, and clinical features with overall mortality in uterine cervical cancer.

	Hazard Ratio	95% CI	*p* Value
Age			
18–30	1.00		
30–45	1.08	0.44 2.67	0.85
45–65	1.29	0.53 3.12	0.54
65–75	1.48	0.61 3.57	0.36
75+	2.23	0.92 5.38	0.07
Year of diagnosis	0.98	0.97 0.99	<0.001
Race			
White	1.00		
Black	1.03	0.94 1.13	0.55
Asian	0.80	0.70 0.91	<0.001
Hispanic	0.84	0.75 0.94	<0.001
Geography			
Metro	1.00		
Adjacent to metro	0.98	0.90 1.06	0.47
Not adjacent to metro	1.09	0.94 1.26	0.32
Unknown	1.32	0.94 1.84	0.10
Yost Index *			
1	1.00		
2	1.02	0.93 1.13	0.60
3	0.98	0.88 1.09	0.78
4	0.91	0.82 1.01	0.13
5	0.92	0.82 1.03	0.18
Charlson Index ^			
0	0.87	0.28 2.71	0.81
1–2	0.27	0.20 0.38	<0.001
3–4	0.39	0.33 0.47	<0.001
5–9	0.68	0.63 0.73	<0.001
10+	1.00		
Histology			
Squamous cell	1.00		
Adenocarcinoma	1.09	1.00 1.19	0.07
Other	1.14	1.02 1.27	0.05
Stage			
Localized	1.00		
Regional	1.89	1.74 2.06	<0.001
Distant	3.80	3.41 4.24	<0.001
MRI			
No	1.00		
Yes	0.95	0.88 1.04	0.25
PET			
No	1.00		
Yes	0.96	0.89 1.04	0.31
CT			
No			
Yes	1.12	1.06 1.23	<0.001
Treated			
No	1.00		
Yes	0.52	0.47 0.56	<0.001

5899 observations were included in the complete case model. * Successive increase in quintile indicates higher socioeconomic status. ^ Higher index is associated with greater morbidity burden.

## Data Availability

Data analyzed in this study were obtained from the SEER-Medicare database (https://healthcaredelivery.cancer.gov/seermedicare/obtain/ accessed 11 October 2026), which is accessible to researchers from the National Cancer Institute after registration and agreement to data use terms.

## Supplementary Materials

The following supporting information can be downloaded at: https://www.mdpi.com/article/10.3390/healthcare14203488/s1, Table S1: Participants with complete versus missing covariate data.
