# Supplementary material for: Uterine Cervical Cancer Treatment and Outcomes Among Medicare-Insured Patients in the United States
Source: Healthcare (Basel). 2026 Oct 17;14(20):3488. doi: 10.3390/healthcare14203488 (PMC13704333; doi:10.3390/healthcare14203488)
Supplement: Supplementary file 1 [file healthcare-14-03488-s001.zip › healthcare-4539032-supplementary.pdf]

Characteristic	Complete Data	Missing Data
	n (%)	n (%)
**Age**		
18-30	39 (0.43)	21 (0.39)
30-45	460 (5.05)	204 (3.75)
45-65	1,866 (20.48)	798 (14.67)
65-75	3,864 (42.41)	2,140 (39.33)
75+	2,882 (31.63)	2,278 (41.87)
**Year of Diagnosis**		
Before 2004	316 (3.47)	3,191 (58.65)
2004+	8,795 (96.53)	2,250 (41.35)

Table S1. Participants with complete versus missing covariate data.
